# Design and Engineering of Semiconductor–Organic Interfaces: Multilayer Synthesis on the Si(001) Surface Based on Cyclooctyne Chemistry

**DOI:** 10.1021/acs.accounts.5c00316

**Published:** 2025-07-23

**Authors:** Michael Dürr, Ulrich Höfer, Ulrich Koert, Ralf Tonner-Zech

**Affiliations:** 1 Institut für Angewandte Physik and Zentrum für Materialforschung, 9175 Justus-Liebig-Universität Giessen, Heinrich-Buff-Ring 16, 35392 Giessen, Germany; 2 Fachbereich Physik, 9377 Philipps-Universität Marburg, Renthof 5, 35032 Marburg, Germany; 3 Fachbereich Chemie, 9377 Philipps-Universität Marburg, Hans-Meerwein-Straße 4, 35032 Marburg, Germany; 4 Wilhelm-Ostwald-Institut für Physikalische und Theoretische Chemie, 200999 Universität Leipzig, Linnéstraße 2, 04103 Leipzig, Germany

## Abstract

In this Account, we will focus on how chemoselective strategies were employed for the controlled synthesis of organic multilayers on silicon, thus forming well-defined semiconductor-organic interfaces. We will start with the chemoselective [2 + 2] cycloaddition of functionalized cyclooctynes to Si–Si dimers which was used as the key step for the surface-orthogonal first layer attachment of bifunctional organic molecules to the Si(001) surface. It results in well-ordered monolayers of bifunctional molecules on the highly reactive Si(001) surface, which serve as a starting point for the further layer-by-layer growth.

Various strategies for the covalent attachment of further molecular layers are then developed; they include, among others, a combination of UHV- and solution-based chemistry for the application of selective and orthogonal reaction schemes in solution as well as a carefully tuned enolether/tetrazine cycloaddition for click chemistry under UHV conditions.

In particular, well-ordered multilayers of organic molecules were realized on prefunctionalized Si(001) via a two-step reaction cycle in solution. Alternating the application of two selective, orthogonal reaction steps based on asymmetric building blocks led to a well-controlled layer-by-layer growth of organic molecular layers on Si(001).

Further exploitation of these strategies, e.g., in the framework of on-surface synthesis, will be discussed; our approach for the selective growth of covalently bound layers perpendicular to the surface might be further combined with lateral structuring of the surface for the synthesis of organic architectures with control in all three dimensions.

## Graphical abstract

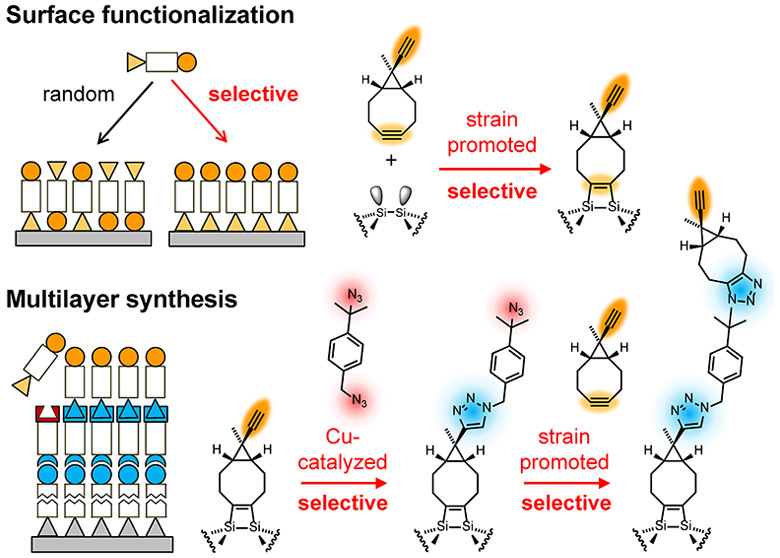

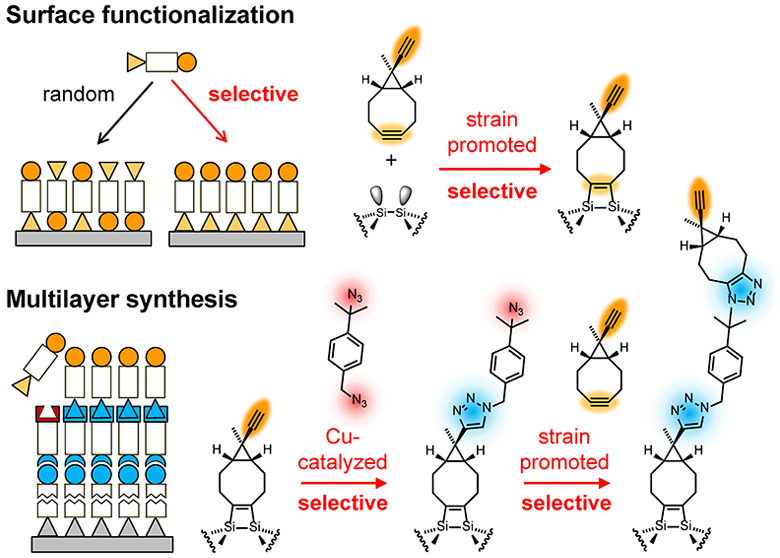

## Key References

-

Reutzel, M. ; Münster, N. ; Lipponer, M. A. ; Länger, C. ; Höfer, U. ; Koert, U. ; Dürr, M. Chemoselective Reactivity of Bifunctional Cyclooctynes on Si(001). J. Phys. Chem. C 2016, 120, 26284–26289 .^1^ *Chemoselective adsorption of substituted (bifunctional) cyclooctynes on Si(001) was demonstrated experimentally. The resulting well-ordered monolayers of organic molecules are the basis for further layer-by-layer growth of organic molecules on silicon.*
-

Münster, N. ; Nikodemiak, P. ; Koert, U. Chemoselective Layer-by-Layer Approach Utilizing Click Reactions with Ethynylcyclooctynes and Diazides. Org. Lett. 2016, 18, 4296–4299 27537081 10.1021/acs.orglett.6b02048.^2^ *The copper­(I)-catalyzed azide–alkyne cycloaddition and the strain-promoted azide–alkyne cycloaddition were shown to be orthogonal click reactions when applied with well-chosen asymmetric bifunctional molecules in solution. Layered model structures were synthesized in solution.*
-

Luy, J.-N ; Molla, M. ; Pecher, L. ; Tonner, R. Efficient Hierarchical Models for Reactivity of Organic Layers on Semiconductor Surfaces. J. Comput. Chem. 2021, 42 (12), 827–839 33617671 10.1002/jcc.26503.^3^ *A hierarchical modeling approach was developed to address the different degrees of complexity in the theoretical description of layer-by-layer synthesis including initial adsorption on the solid surface and further growth of the subsequent molecular layers.*
-

Glaser, T. ; Peters, J. A. ; Scharf, D. ; Koert, U. ; Dürr, M. Layer-by-Layer Deposition of Organic Molecules Controlled by Selective Click Reactions. Chem. Mater. 2024, 36, 561–566 .^4^ *Well-controlled layer-by-layer synthesis of organic structures on Si(001) was realized by a combination of UHV-based functionalization of the Si(001) surface using bifunctional cyclooctynes and further layer-by-layer growth in solution using two orthogonal click reactions based on asymmetric bifunctional molecular building blocks.*

## 1 Introduction

Organic materials are of growing importance in electronic devices. Charge transfer processes across semiconductor/organic interfaces are of particular importance for such hybrid devices, e.g., in optoelectronic applications. The preparation of these interfaces with high chemical precision is thus of interest for semiconductor-based applications; they can also serve as model systems with defined structural and electronic properties.

Various methods are available for the well-defined preparation of organic layers on semiconductor surfaces, such as physical vapor deposition,^5^ molecular beam epitaxy,^6,7^ chemical vapor deposition,^8–10^ or atomic layer deposition.^10–13^ For van-der-Waals-bound molecular layers, physical vapor deposition or the preparation from solution are typically applied.^14^ For covalently bound films, molecular layer deposition (MLD),^15–18^ which follows a molecular layer-by-layer (LBL) synthesis, is often used. It relies both on the covalent fixation of the organic molecules to the inorganic semiconductor surface and on the covalent attachment of the further layers (Figure 1(a)).

**Figure 1:**
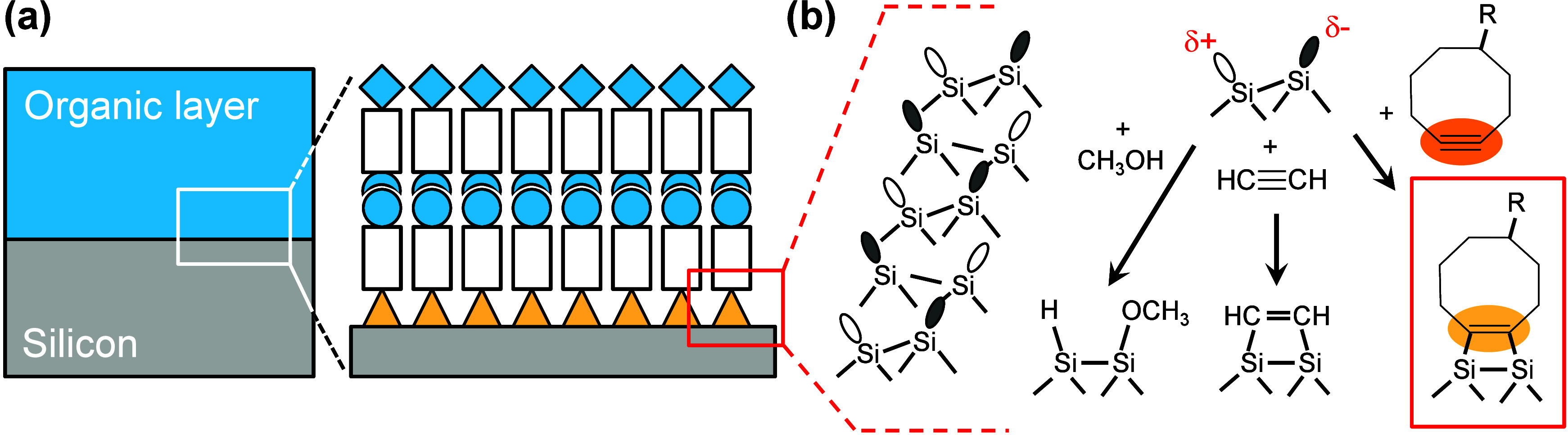
(a) Schematic depiction of an inorganic/organic interface on silicon based on the direct (covalent) fixation of the organic molecules to the inorganic silicon substrate. (b) Si(001) surface reconstruction and typical reactions with organic molecules.

When it comes to the application of LBL synthesis of inorganic/organic interfaces on the technologically most important semiconductor surface, i.e., on Si(001) (Figure 1(b)), one major challenge is given by the high reactivity of this surface: Despite the comparably simple reconstruction of Si(001) in rows of silicon dimers (Figure 1(b)), it displays a broad variety of different chemical reactions with organic functional groups.^19–22^ The electronic structure of each silicon dimer can be approximated by the local description of one unfilled dangling bond (δ+) and one dangling bond filled with two electrons (δ−), which determine the chemical reactivity of the surface. Typical chemical reactions of organic molecules with these silicon dimers are dissociative reactions, e.g., dissociative adsorption of H_3_CO-H,^23–30^ and [2 + 2] cycloaddition to pi-bonds resulting in four-membered rings^19,20,31–34^ (Figure 1(b)). They all proceed with high reactivity which typically prohibits chemoselective addition of asymmetric bifunctional building blocks to the surface (Figure 2(a)).^21^ However, such chemoselective adsorption of bifunctional molecules is the prerequisite for the formation of the first organic layer on Si(001) which then serves as the starting point for directed vertical molecular LBL growth (Figure 2). With respect to this first challenge, we show in the following that functionalized cyclooctynes adsorb chemoselectively via cycloaddition of the strained triple bond to the silicon dimer (Figures 1(b) and 2(a)).

**Figure 2:**
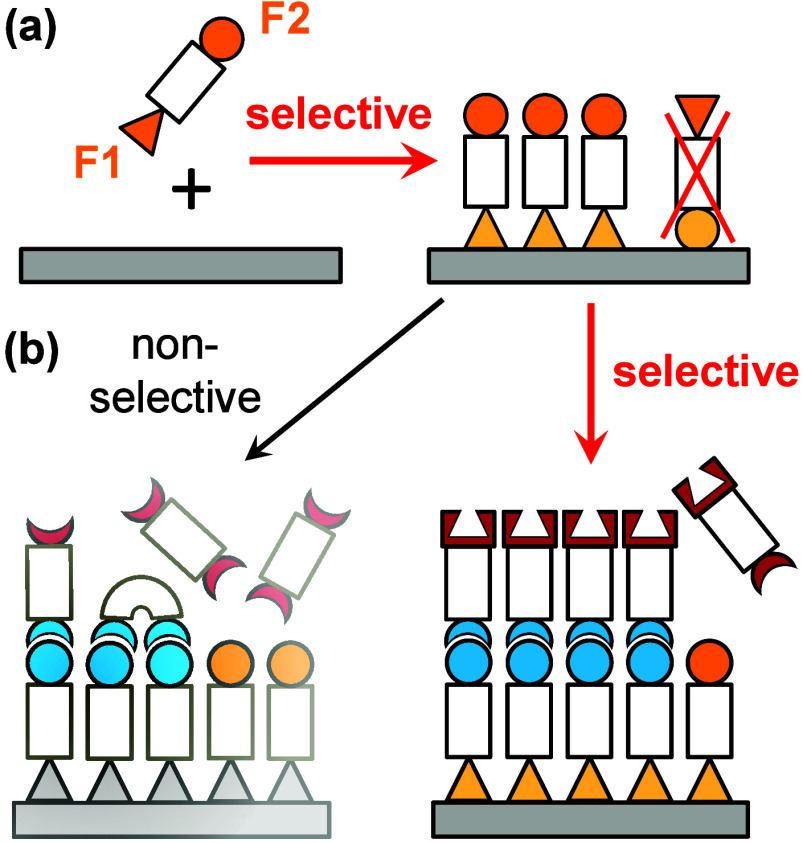
(a) Chemoselective adsorption of bifunctional organic molecules on Si(001) is required for the attachment of the first organic layer of well-ordered inorganic/organic interfaces. (b) Selective reactions of bifunctional molecules are also required for the attachment of further layers (right) in order to avoid side reactions such as chain termination (left).

The second challenge that we addressed was the design of a reaction sequence for the synthesis of the subsequent layers. We developed a strategy that is based on selective reactions for each layer formation step. Such a selective approach should then lead to a most well-defined LBL attachment of the further molecular layers with a minimum of side reactions which are typically operative when using symmetric building blocks (thus nonselective reactions) in molecular layer deposition (Figure 2(b)).^35,36^ Furthermore, the reaction sequence for the synthesis of the subsequent layers should be compatible with the preparation of the functionalized silicon surface under ultrahigh vacuum (UHV) conditions.

## 2 Selective Cyclooctyne Chemistry for LBL Synthesis

A vertical and flexible molecular layer-by-layer synthesis at the Si(001) surface requires bifunctional building blocks with orthogonal reactivity. Functionalized cyclooctynes are promising building block candidates for orthogonal reactivity because their high-energetic strained triple bond^37^ can be used for chemoselective reactions. The remaining second functionality is then available for the covalent attachment of the subsequent molecular architecture. This concept was applied successfully with the bioorthogonal use of cyclooctynes in chemical biology.^38^ In addition, substituted cyclooctynes could also be used for the selective functionalization of the Si(001) surface in a surface-orthogonal mode (see below).

Compared to the various analytical techniques for solution synthesis (NMR, X-ray), the analytical methods for on-surface chemistry are more restricted. It was therefore advisable to develop the LBL cyclooctyne chemistry in solution first. Chemistry under UHV-only conditions typical for Si(001) surface studies has to work without additives or catalysts as used in solution chemistry. An “additive-free“, very efficient reaction is the inverse-electron-demand Diels–Alder (IEDDA) cycloaddition of a tetrazine with an electron rich alkene.^39^ The tetrazine diester **1** bears an azide in the side chain (Figure 3(a)). It is capable of performing two reactions: an inverse-electron-demand Diels–Alder (IEDDA) tetrazine-enol-ether cycloaddition and a strain-promoted azide alkyne cycloaddition (SPAAC).^38^ Its complementary reaction partner is the cyclooctyne enol ether **2**.^40^ A one-dimensional LBL-model synthesis was developed that started with the strain-promoted Diels–Alder addition of **2** to the pyrone **3** and a subsequent retro-Diels-Alder produced the enol ether **4** (Fig. 3(b)). In this solution model system, the benzene ring in **4** mimics the latter semiconductor surface. IEDDA reaction of **4** with **1** provided the pyridazine **5**. Subsequent SPAAC of **5** with **2** leads to **6**. Thus, the sequential use of IEDDA and SPAAC results in a defined predictable chain growth in this one-dimensional model system.

**Figure 3:**
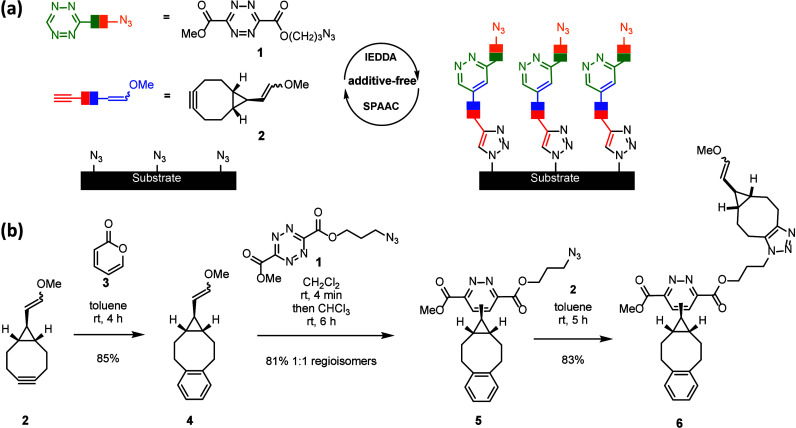
(a) Selective coupling via enolether/tetrazine cycloaddition (IEDDA) and azide/alkyne coupling via SPAAC. (b) One-dimensional solution model system. Reproduced from ref 40. Copyright 2019 American Chemical Society.

The copper­(I)-catalyzed azide–alkyne cycloaddition (CuAAC) that produces triazoles is so efficient and powerful that the term click reaction was coined for it.^41^ Its use for molecular layer-by-layer synthesis on surfaces thus seems to be a natural choice but so far, it had been limited mainly to symmetrical bifunctional building blocks (symmetrical diazides and dialkynes).^42^ On the other hand, with the strain-promoted azide alkyne cycloaddition (SPAAC), another highly efficient click reaction is available within the same family of reactions which furthermore makes use of cyclooctyne, the main building block of our strategy for controlled surface functionalization. Toward the goal of a vertical LBL-synthesis, we thus designed and synthesized the building blocks **7** and **8** (Figure 4(a))^2,43^ for a *sequential combination of CuAAC and SPAAC*. Both molecules contain the same functional group twice, but with a predictable order of reactivity which ensures chemoselectivity: in **7**, the strain-promoted cyclooctyne is more reactive than the α-disubstituted terminal alkyne and in **8** the primary azide reactivity dominates over the tertiary azide. A “molecular surface” was used as model system for the silicon surface in order to develop and optimize the CuAAC/SPAAC sequence in solution. The cholic acid derived triazide **9** ^44^ was chosen as such a model system (Figure 4(b)). A 3-fold SPAAC-reaction with the bisalkyne **7** led to the tristriazole **10** with very good chemoselectivity and excellent yield. The subsequent 3-fold CuAAC-reaction with diazide **8** installed the next triazole layer with very good efficacy and selectivity. Another threefold SPAAC-reaction with the bisalkyne **7** produced the third layer in form of compound **12**. These results proved the sequential combination of CuAAC and SPAAC using building blocks **7** and **8** as a successful strategy and paved the way to an LBL-synthesis on the silicon surface. However, this enterprise would require a unified approach using UHV and solution chemistry.

**Figure 4:**
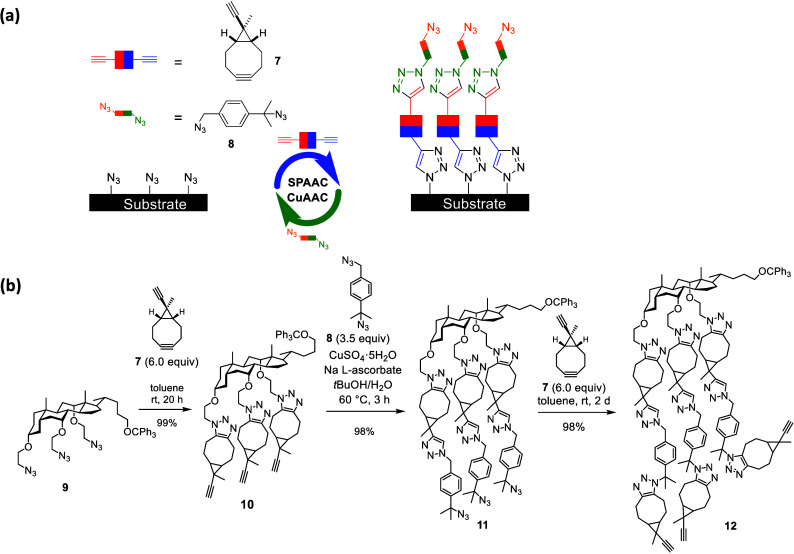
(a) Selective LBL-synthesis via sequential strain promoted and Cu­(I)-catalyzed azide/alkyne reactions in solution. (b) Sequential combination of CuAAC and SPAAC for LBL-synthesis in solution using triazide **9**. Reproduced from ref 2. Copyright 2016 American Chemical Society.

## 3 Chemoselective Reactivity of Organic Molecules on Si(001) Based on Functionalized Cyclooctynes

The high reactivity of the Si(001) surface toward organic functional groups typically leads to random adsorption of bifunctional organic molecules on Si(001), i.e., adsorption takes place via one or the other or even via both functional groups (Figure 2(a)). However, the vast majority of these functional groups reacts on Si(001) via a weakly bound intermediate state;^19,21,22^ further reaction from this intermediate into the covalently bound final state proceeds via an energy barrier in the range of typically 0.1 to 0.4 eV.^32,46–51^ As an example, a schematic energy diagram for the adsorption of acetylene on Si(001) (Figure 5(a)) is shown in the inset of Figure 5(b). The addition of acetylene to dimer **13** proceeds via intermediate **14** and leads to cycloadduct **15**. As a result of this indirect reaction channel, the sticking probability of acetylene decreases with increasing surface temperature (Figure 5(b)): from the intermediate, the weakly bound molecule can either convert into the final state or desorb back into the gas phase. At increased surface temperature, the latter process is of more importance, leading to the decreasing sticking probability. In contrast to this behavior and thus in contrast to almost all other organic functional groups, cyclooctyne with its strained triple bond shows a constant sticking probability on Si(001), independent of surface temperature (Figure 5(b)). This indicates a direct reaction channel for cyclooctyne **16** on Si(001) without an intermediate (inset of Figure 5(b) and Figure 5(c)).

**Figure 5:**
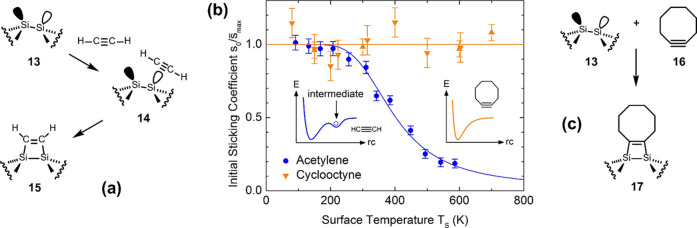
(a) Reaction pathway of acetylene on Si(001). (b) Normalized initial sticking coefficients *s*_0_ for acetylene (blue dots) and cyclooctyne (orange triangles) on Si(001) as a function of the surface temperature. The blue line in the main panel represents the quantitative description of the data by the Kisliuk precursor model.^45,46^ (c) Reaction pathway of cyclooctyne on Si(001) without an intermediate. Reproduced with permission from ref 47. Copyright 2019 The Institute of Physics.

This difference in the energetics of the adsorption channels is the basis for the chemoselective adsorption of substituted cyclooctynes on Si(001) as illustrated in Figure 6 for ethynyl cyclopropyl cyclooctyne (ECCO, **7**) adsorbed on Si(001). The broad line in the C 1s spectrum (Figure 6(a)) depicts a clear shoulder at lower binding energies indicating Si–C bond formation.^1,28,52,53^ Careful analysis of the line profile revealed an intensity ratio between this component and the other contributions of 1:5 indicating adsorption of the ECCO molecule via one of the two triple bonds only. STM images of ECCO adsorbed in Si(001) (Figure 6(b)) revealed one single adsorption configuration that was restricted to one dimer row. In comparison, acetylene adsorbs on Si(001) in two different configurations, which both do not fit the configuration observed in Figure 6(b); the STM images were thus interpreted in terms of selective adsorption via the strained triple bond of cyclooctyne only.^47^ From the symmetric shape of the features with respect to the dimer rows, adsorption on top of one dimer (**18**) was concluded (Figure 6(c)).

**Figure 6:**
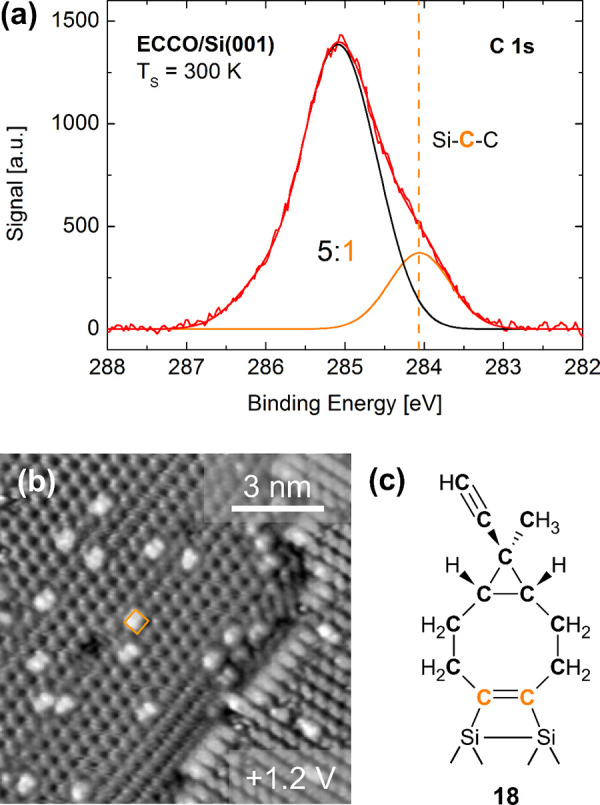
(a) C 1s core level spectrum measured after the adsorption of ECCO **7** on Si(001) at 300 K. (b) Empty state STM image of 0.03 ML ECCO on Si(001) adsorbed and measured at 50 K. (c) Sketch of ECCO adsorbed on-top of one Si dimer via the strained triple bond of the cyclooctyne ring. Reproduced with permission from ref 47. Copyright 2019 The Institute of Physics.

Chemoselective adsorption of substituted cyclooctynes was demonstrated not only for bisalkyne **7** but also for cyclooctyne ester **19** and ether **20**,^1,54^ as well as cyclooctyne enol ether **2** ^55^ (Figure 7).

**Figure 7:**
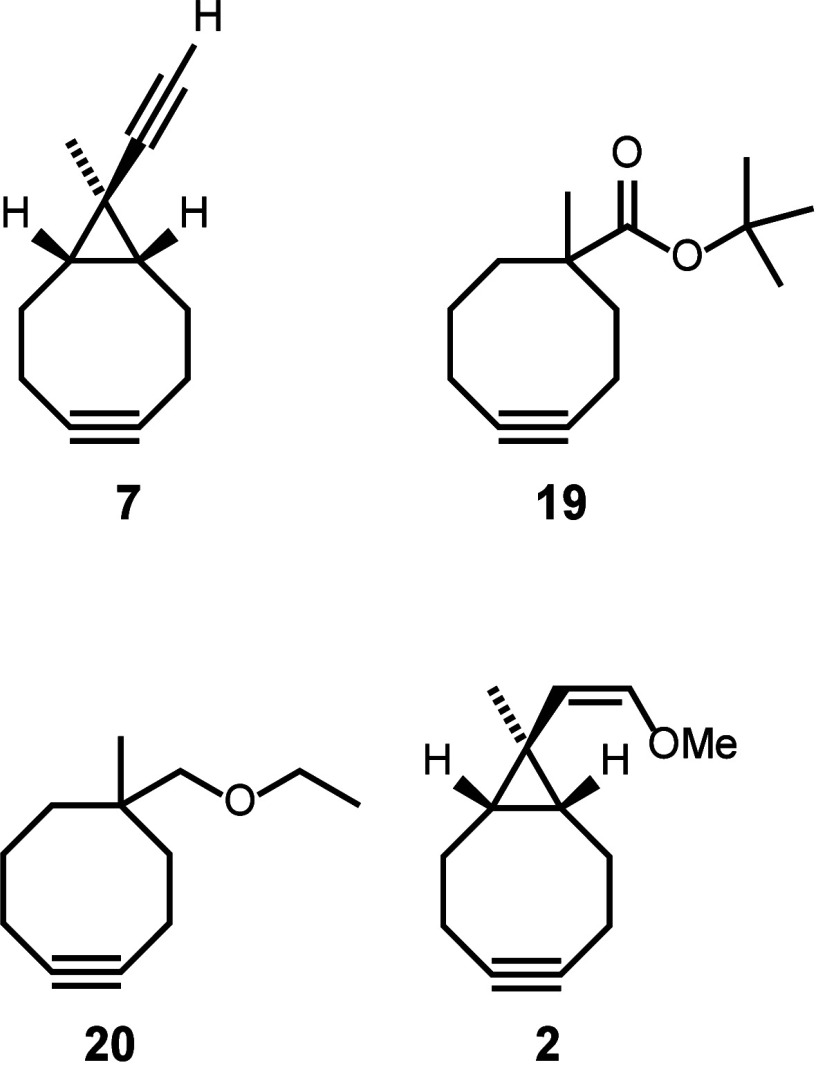
Structures of bifunctional cyclooctynes **7**, **19**, **20**, and **2** that show chemoselective cycloaddition to the Si(001) surface.

The observed chemoselective adsorption of substituted cyclooctynes was explained on the basis of the two different types of reaction channels involved: if the substituted cyclooctyne approaches the surface with the strained triple bond (F1 in Figure 2(a)), then the molecule reacts via the direct reaction channel into the covalently bound final state. If the substituted cyclooctyne approaches the surfaces with the second (additional) functional group (F2 in Figure 2(a)), it might be first trapped in the intermediate adsorption state formed by the second functional group and the dangling bonds of the silicon surface. As these intermediates are comparably long-lived (e.g., ≈100 ms for Et_2_O at 300 K^49^), the molecule has enough time to probe the surface with its strained triple bond. Apparently, this leads to [2 + 2] cycloaddition of the cyclooctyne along with the release of the weak bond forming the intermediate state thus finally resulting in a molecule solely bound via the cyclooctyne unit.

This qualitative reasoning was backed by computational investigations of the adsorption pathways of cyclooctyne and substituted cyclooctynes. They started from parent cyclooctyne to derive a static and dynamic picture of bonding and reactivity.^56,57^ The strained triple bond was found to be the decisive feature of cyclooctyne to enable a selective adsorption on-top of the silicon dimer. The reduced energy to deform the molecule for surface bonding (preparation energy) is key for the stronger bonding compared to acetylene as found from an analysis of the bonding contributions with an energy decomposition analysis for periodic systems (pEDA).^56,58^ The direct adsorption pathway observed in experiment posed significant challenges for the computational modeling. Static DFT computations for the reaction path toward the surface could not reveal the precursor state. Only the application of ab initio molecular dynamics (AIMD) methods revealed the pseudodirect adsorption pathway with an ultrashort-lived intermediate state which converts toward the [2 + 2] cycloaddition product via a negligible reaction barrier.^59^

In a first step toward the analysis of bifunctional building blocks, the adsorption behavior of 5-ethoxymethyl-5-methylcyclooctyne (EMC, **20**) was investigated.^60^ Although this system was not a target for the LBL synthesis, it provided an opportunity for the systematic analysis of competitive surface bonding of two different functional groups since the ether reactivity on Si(001) had been systematically analyzed before.^52,61–63^ It was shown that the two functional groups react independently of each other and the strained triple bond is the more reactive one toward the surface (Figure 8) in agreement with experiment.^1^ Notably, the bonding nature of the triple bond toward the surface as analyzed by pEDA is virtually equal to parent cyclooctyne as expected from a nonconjugated hydrocarbon ring; the only difference is a higher dispersion attraction toward the surface by the second functional group. To target these large and complex molecules interacting with the surface, a hierarchical modeling approach had been developed,^3^ which was used, e.g., in the modeling of the enolether coupling reaction (compare Figure 9).

**Figure 8:**
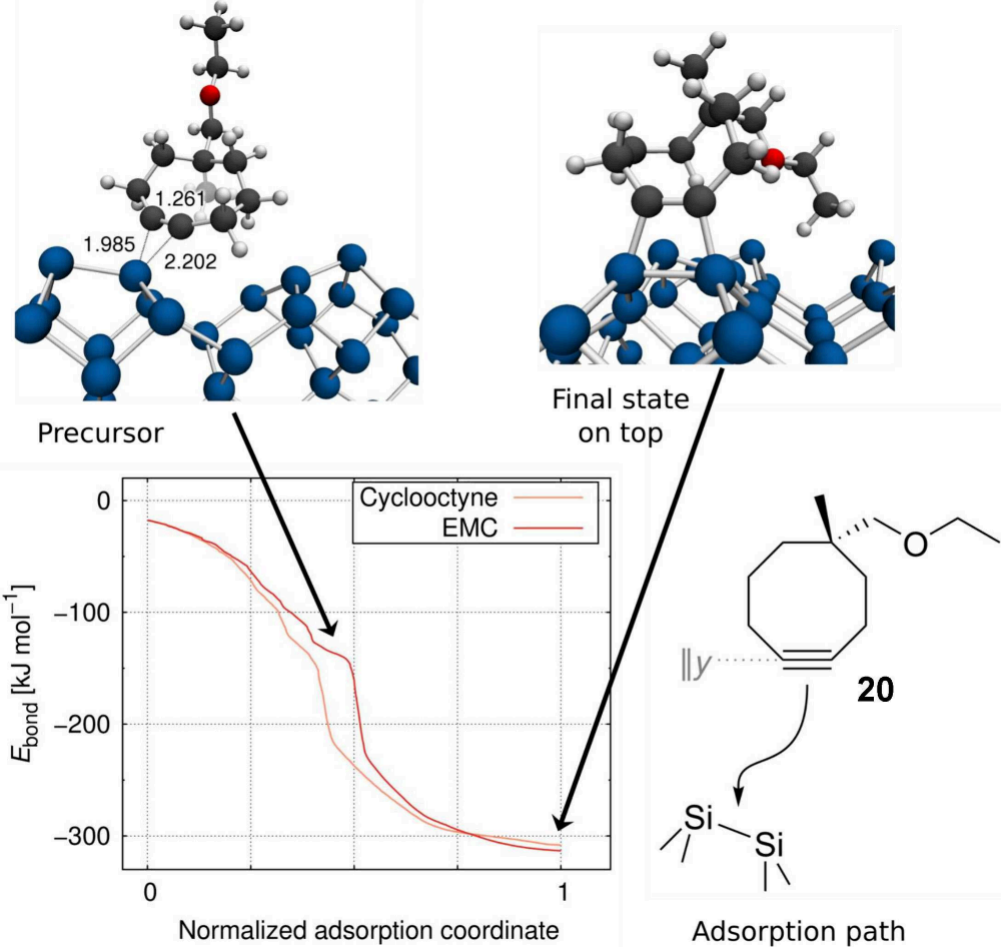
Adsorption dynamics of EMC from DFT: Computed adsorption pathway of parent cyclooctyne and bifunctional EMC. Both molecules follow the same pseudodirect reaction channel via a ultrashort-lived precursor state (upper left) into the final [2 + 2] cycloadduct on-top of one silicon dimer (upper right). Lower right: Schematic adsorption pathway of EMC on Si(001). Adapted from ref 60. Copyright 2018 American Chemical Society.

**Figure 9:**
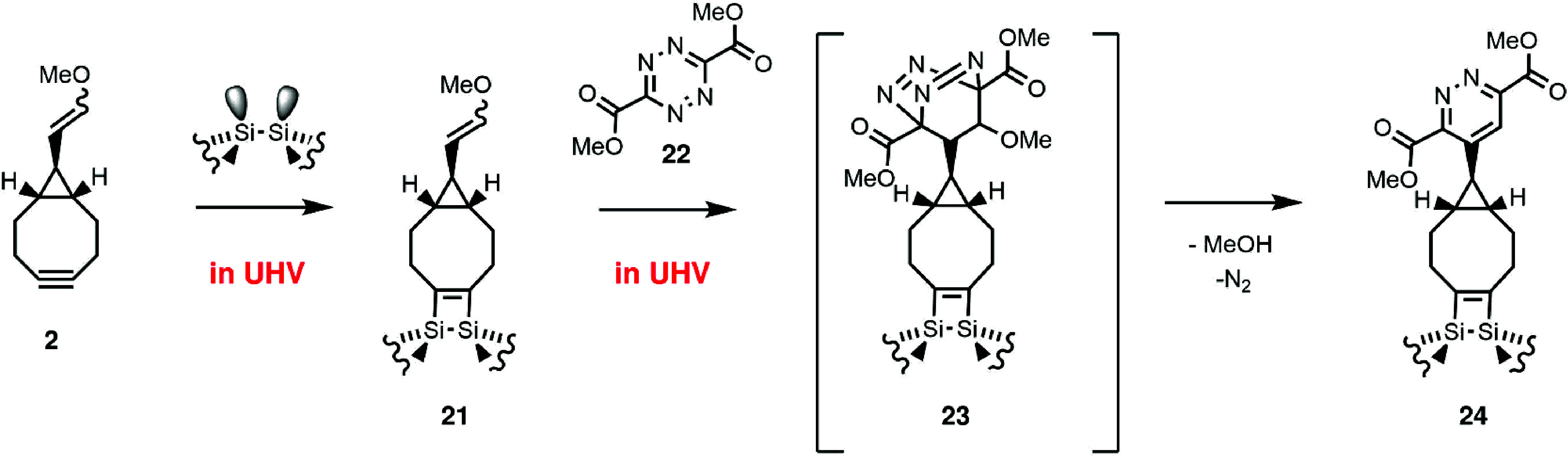
Enolether/tetrazine coupling for multilayer synthesis on Si(001): MEECO (**2**) reacts on the dimers of Si(001) via the strained triple bond of cyclooctyne forming **21**.^55^ 1,2,4,5-Tetrazine-3,6-dicarboxylate (**22**) reacts with **21** via **23** to the final product **24** releasing N_2_ and MeOH.^65^ Reproduced from ref 65. Available under a CC BY-NC-ND 4.0 license. Copyright 2021 The authors.

The now established methodology was then applied to ECCO adsorbing on Si(001).^64^ The combination of static DFT investigations, bonding analysis via pEDA, and AIMD methodology revealed again a preference for the strained triple-bond of the cyclooctyne ring in adsorption to the surface when compared to the unstrained acetylene group. pEDA revealed that the lower preparation energy is the decisive term, in agreement with the analysis of the separate systems discussed above. In order to reach sufficient statistical sampling of the computationally demanding AIMD runs, machine-learning accelerated molecular dynamics approaches were applied, which allowed for the computation of 10.000 trajectories for ECCO adsorbing on silicon. The resulting distribution shows a clear preference for the singly bonded structures, in line with the experimental findings.

## 4 Synthesis of Organic Multilayers on Silicon

### 4.1 Click Chemistry on Functionalized Silicon in UHV

Following the results obtained for solution-based cyclooctyne chemistry (section 3), enolether/tetrazine cycloaddition was identified as a promising candidate for coupling a second layer of organic molecules to the cyclooctyne-functionalized Si(001) surface under UHV conditions. The reaction scheme is outlined in Figure 9: First, methyl enolether-substituted cyclooctyne (MEECO, **2**) is added chemoselectively to the surface to produce the [2 + 2] cycloadduct **21**. The second layer is then covalently constructed by [4 + 2] cycloaddition of the enol ether functionality with dimethyl 1,2,4,5-tetrazine-3,6-dicarboxylate (**22**) which leads via intermediate **23** and its eliminative aromatization to pyridazine **24**. In solution, this reaction sequence proceeds at room temperature without catalyst and with high yield (section 3).^66^ Computational results combining periodic DFT calculations with correction schemes based on highly accurate CCSD­(T) approaches showed the reaction pathway in vacuum to proceed via a finite energy barrier of Δ*G*(298 K) = 71 kJ/mol (with respect to the reactants) associated with the formation of the intermediate **23** from **21** (Figure 9).^65^ In the further course of the reaction toward the final pyridazine product **24**, methanol and N_2_ are released, which contributes together with the rearomatization to the strong thermodynamic driving force calculated for the overall reaction (Δ*G*(298 K) = 394 kJ/mol).^65^ Experimentally, the MEECO-covered surface **21** was the starting point.^55^ Product **24** was only detected when tetrazine **22** was dosed at a surface temperature of 380 K, indeed indicating the activated nature of this process.^65^

### 4.2 Combination of UHV- and Solution-Based Chemistry

While the UHV-based enolether/tetrazine cycloaddition (IEDDA) LBL-approach presented in Sec. [Sec sec5.1] requires a well-tuned temperature control for the retro [4 + 2] cycloaddition to generate the pyridazine, the solution-based CuAAC-route works completely at ambient temperature. The solvent in the CuAAC-route may have several advantages compared to UHV-chemistry: it allows the use of homogeneous catalysts such as Cu­(I), helps with transport and detransport of reactants, and supports transition states. Thus, for a higher flexibility with respect to the further reaction steps and in order to make use of the advantages of solution chemistry, the application of CuAAC-chemistry for anchoring the second organic layer on the cyclooctyne functionalized Si(001) surface was undertaken.

The concept is outlined in Figure 10(a): the first organic layer on Si(001) is prepared with atomic precision under UHV conditions by chemoselective adsorption of substituted cyclooctynes, e.g., ECCO. After analysis by means of XPS, the sample is directly transferred into a dedicated reaction chamber equipped with stainless steel beakers filled with the respective solutions and solvents (Figure 10(c)). In our first example, we used methyl-substituted benzyl azide **25** (Figure 10(b)) in acetonitrile for Cu-catalyzed azide–alkyne cycloaddition to attach the second organic layer to the ECCO-covered Si(001) surface **18**.^67^ CuBr­(PPh_3_)_3_ was used as the catalyst. After a given time in solution and rinsing the sample in the bare solvent, the sample was transferred back into UHV and reaction product **26** was analyzed by means of XPS again. In order to avoid collateral damage of the precious UHV-apparatus due to, e.g., chemical side reactions of the copper joints, preliminary experiments revealed that CuBr­(PPh_3_)_3_ and acetonitrile caused no harm.

**Figure 10:**
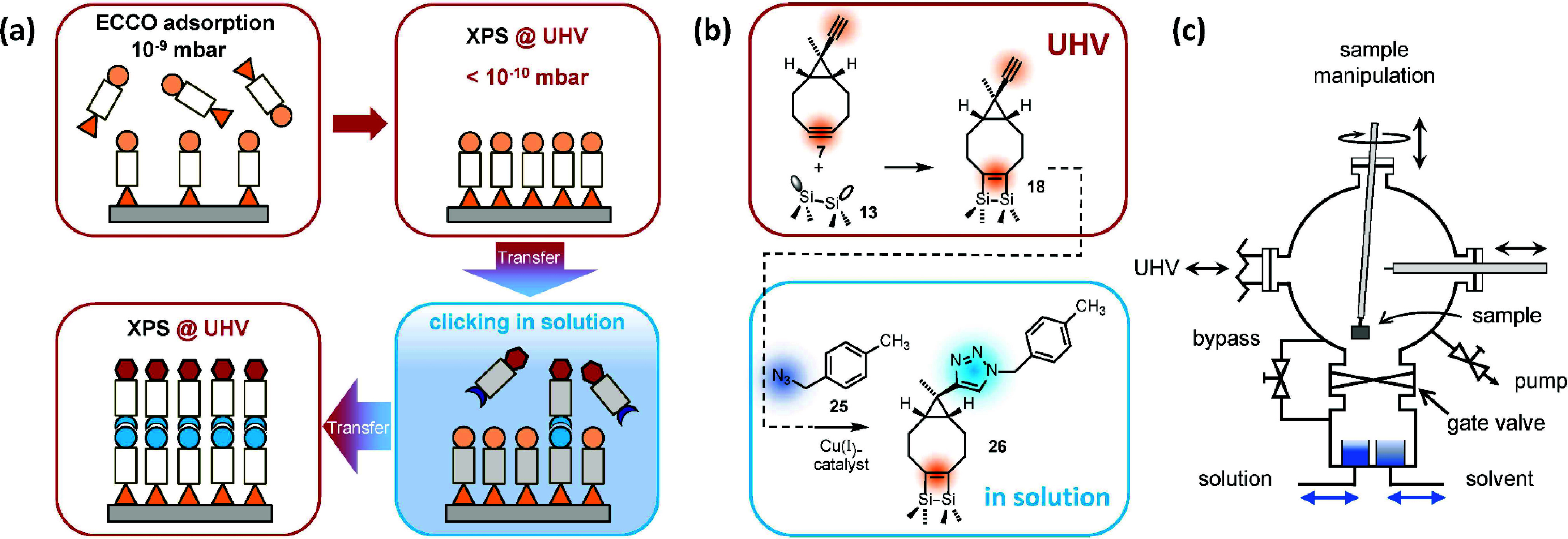
Combination of UHV- and solution-based chemistry for the growth of organic layers on Si(001). (a) Schematic representation of the experiment. (b) Reaction schemes employed. (c) Schematic drawing of the dedicated reaction chamber. Reproduced from ref 67. Copyright 2021 American Chemical Society.

The experimental results are summarized in the N 1s core level spectrum, shown in Figure 11(b). Whereas no nitrogen signal was detectable from the ECCO-covered Si(001) surface after transfer into liquid acetonitrile in the reaction chamber, a clear signal was observed when the ECCO-functionalized Si surface was kept for 60 min in the azide acetonitrile solution (*c*_azide_ = 0.5 mol/L, *c*_catalyst_ = 5 × 10^–3^ mol/L). The three main components between 400 and 403 eV (Figure 11(b)) can be assigned to the click reaction product, the 1,4-disubstituted triazole **26** (Figure 10(b)) with the nitrogen atoms being part of the triazole ring.^69–72^ Furthermore, no signal intensity is observed at 405 eV. As the latter is indicative of the intact azide group (Figure 11(a)^67,69,70,73^ ), one can conclude that no intact benzyl azide molecules contribute to the N 1s signal intensity shown in Figure 10(b). Thus, the vast majority of the benzyl azide molecules were attached via the click product **26** with only a small number of benzyl azide molecules directly attached to the silicon substrate (component at 398 eV); the small intensity at 399 eV was interpreted as the consequence of the remainders of the catalyst on the surface. The respective C 1s spectrum was also in agreement with product **26**.^67^

**Figure 11:**
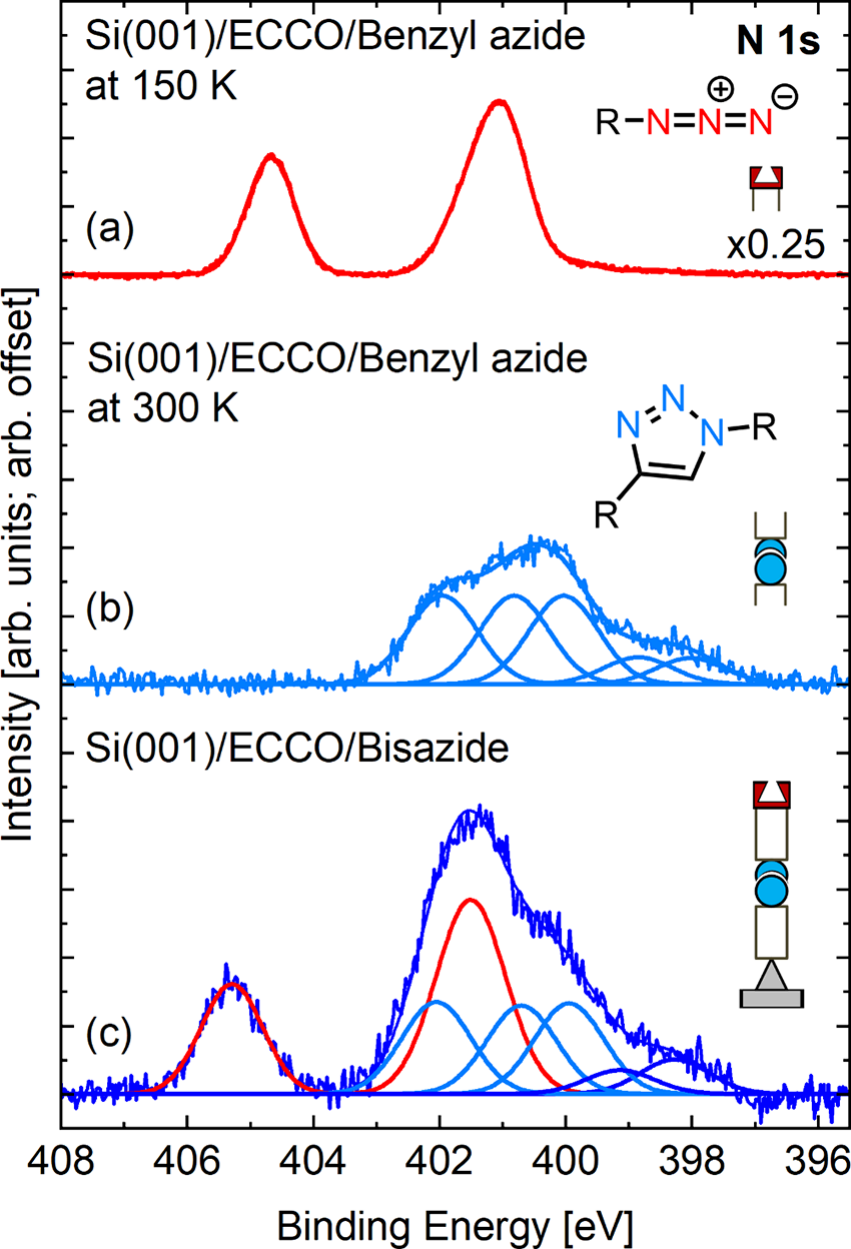
N 1s core level spectra (a) of intact benzyl azide condensed on ECCO-functionalized Si(001) at *T*_S_ = 150 K.^68^ (b) After reaction of benzyl azide with ECCO-functionalized Si(001) indicative of the triazole ring as part of the reaction product. (c) After reaction of diazide **8** with ECCO-functionalized Si(001) **18**. Reproduced from ref 4. Copyright 2024 American Chemical Society.

### 4.3 Application of Orthogonal Click Reaction Schemes for LBL Growth

When compared to the attachment of the second layer only (sections 5.1 and 5.2), bifunctional molecules have to be used for each of the layers in multiple LBL synthesis. However, the use of symmetric bifunctional molecules can lead to side reactions such as chain termination, ultimately leading to the termination of the LBL growth (Figure 12(a), “non-selective route” ^35,36^ ).

**Figure 12:**
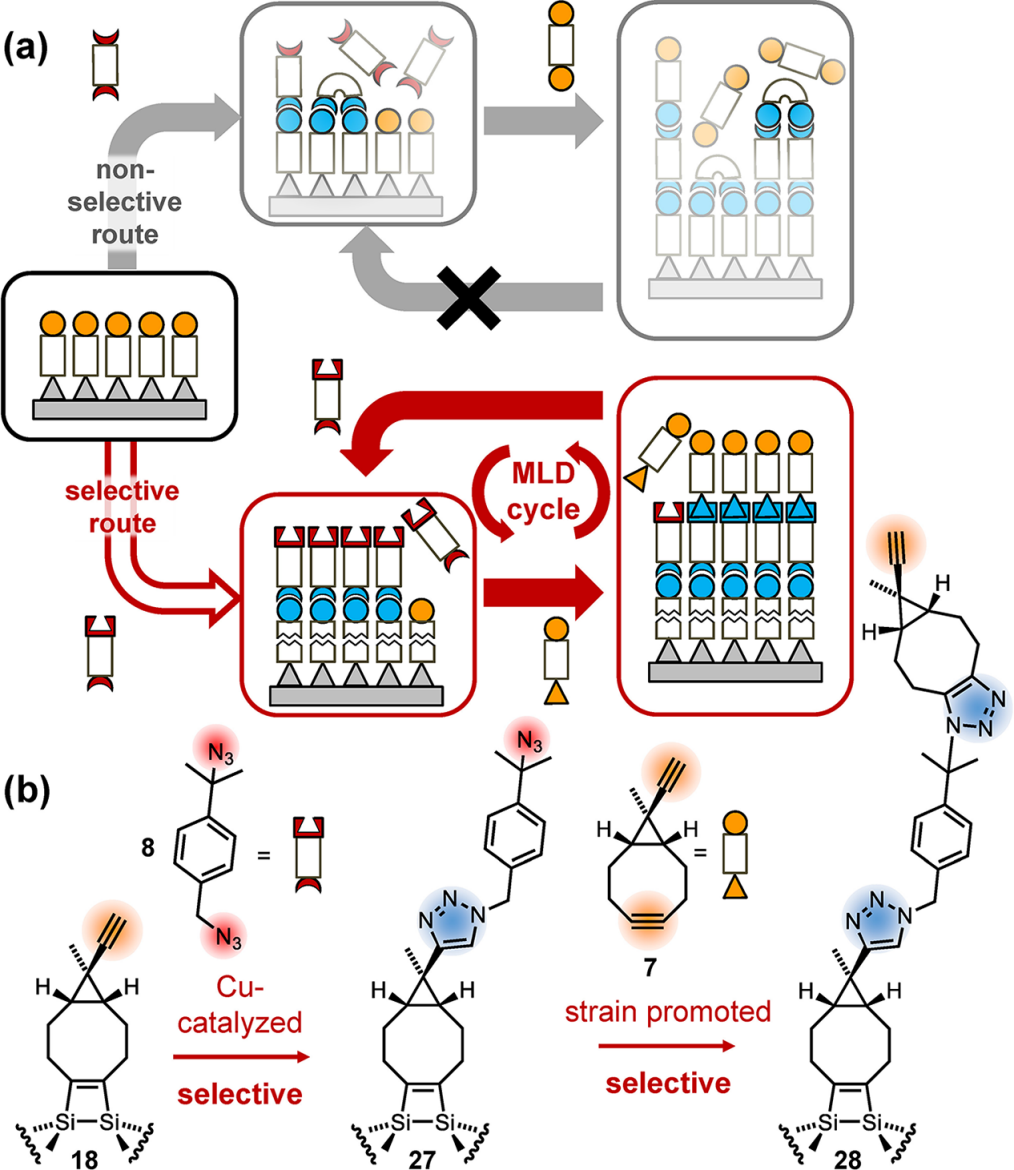
Schematics of the layer-by-layer synthesis of organic films on Si(001). (a) Nonselective route (symmetric building blocks) versus selective route (asymmetric building blocks). (b) Reaction schemes employed for the selective route. Reproduced from ref 4. Copyright 2024 American Chemical Society.

In analogy to the results described in section 3, we thus employed two selective, orthogonal reaction steps based on two asymmetric bifunctional building blocks (Figure 12(a), “selective route”):^2,4^ for the second layer, 1-(azidomethyl)-4-(2-azidopropan-2-yl)­benzene (**8**), short diazide, is used. Only the primary azido group reacts selectively with the terminal triple bond of surface-adsorbed ECCO **18** (Figure 12(b)). In this reaction step, the tertiary azido group in product **27** stays intact and can be used for the attachment of the third layer (**27** + **7** → **28**). The strained triple bond of cyclooctyne has been shown to readily react with the tertiary azido group at room temperature even without catalyst (section 3).^2^ Thus, when ECCO is used for building the third layer, the strained triple bond of cyclooctyne reacts selectively with the tertiary azido group of the second layer, leaving the terminal triple bond of ECCO intact for the attachment of the fourth layer. If diazide **8** is used for this fourth layer, a second cycle of the layer-by-layer synthesis is started and the alternating application of the two orthogonal reaction steps will lead to the growth of well-ordered organic multilayers on Si(001).

Experimentally, the reaction products were analyzed by means of XPS after each of the reaction steps. In Figure 11(c), the spectrum obtained after reaction of the ECCO-functionalized Si(001) surface in diazide acetonitrile solution (*c*_diazide_ = 0.5 mol/L, *c*_catalyst_ = 5 × 10^–3^ mol/L) is shown. The signal intensity at 405 eV indicates the presence of intact azido groups (represented by the components shown in red); the additional intensity in the energy range between 399 and 403 eV further indicates the presence of triazole rings (represented by the components shown in blue). The total spectrum shown in Figure 11(c) can be perfectly fitted by a 1:1 superposition of the spectra shown in Figures 11(a) and (b) thus indicating selective attachment of the diazide molecules via the primary azido group only.

Attachment of the third layer of organic molecules was then realized via an in-solution reaction with ECCO (*c*_ECCO_ = 0.5 mol/L, in acetonitrile, no catalyst). In the XPS spectrum taken of the third layer (Figure 13(a)), no signal intensity at 405 eV is observed. This indicates the reaction of the tertiary azido group of the second layer with the strained triple bond of ECCO (Figure 12(b)); the terminal triple bond does not react with the tertiary azido group under the given conditions and stays intact for further reactions. The resulting spectrum can be again perfectly fitted with the spectrum known from the triazole ring (Figure 11(b)).

**Figure 13:**
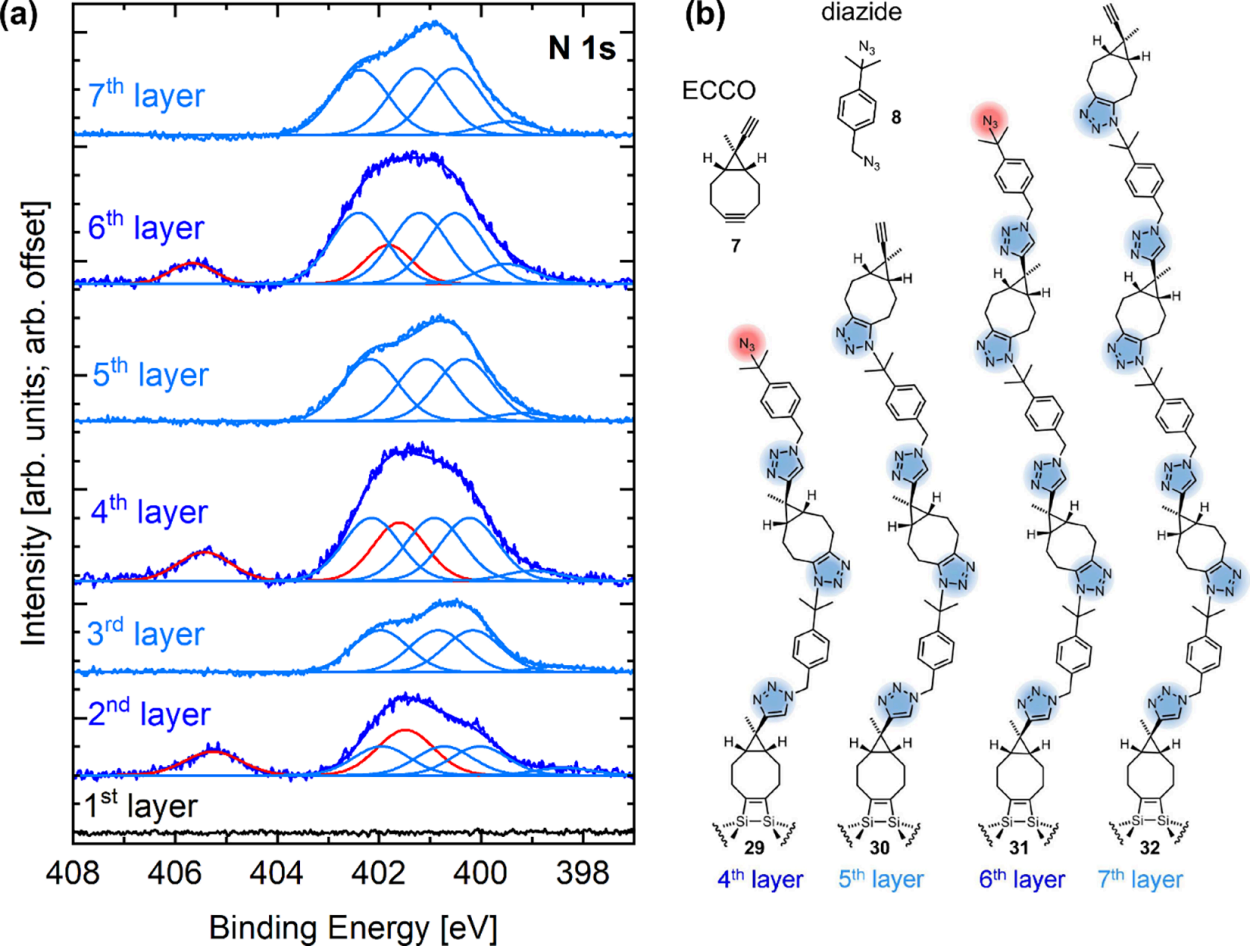
(a) N 1s spectra after different reaction steps on the Si(001) sample. First layer: After ECCO adsorption. In the second, fourth, and sixth layer, attachment of diazide molecules led to the peaks in red indicating intact azido groups (405 eV) in the top layer; after every additional reaction step in ECCO solution (third, fifth, and seventh layer), no intensity at 405 eV is observed and the total intensity can be described by the components assigned to the triazole ring. (b) Molecular structures of the respective organic multilayers. Reproduced from ref 4. Copyright 2024 American Chemical Society.

With the attachment of the further layers, we observe alternatingly the appearance of the azido-signal intensity at 405 eV when the even-numbered layers are attached and its disappearance when the odd-numbered layers are attached (Figure 13(a)). The spectra of the odd-numbered layers are perfectly represented by the components found for the triazole ring; the spectra of the even-numbered layers are a superposition of the components of the intact azido group and triazole ring. The sequence of spectra in Figure 13(a) thus demonstrates the alternating attachment of diazide and cyclooctyne molecules as illustrated in Figure 13(b). From the quantitative evaluation of the C 1s and Si 2p signal intensity with increasing number of cycles, an average layer thickness of 1 nm was deduced, in good agreement with the size of the molecules.^4^

Further quantification yielded about 70% of the dangling bonds being saturated by ECCO in the first layer, given by the average surface area covered by one cyclooctyne molecule, which is larger than one dimer.^74^ 70% of these ECCO molecules are then reacted by the azide molecules of the second layer; this further reduction of the molecular density per dimer might be also interpreted in terms of the finite surface area accessible for the azide molecules and in part by steric hindrance of the terminal triple bonds. The yield for the attachment of the molecules in the third and further layers is then close to 100%. In part, this is attributed to the flexibility of the longer molecular chains in solution, which geometrically facilitates the strain-promoted click reaction to take place.

## Conclusion and Outlook

In conclusion, we successfully combined synthetic organic chemistry with semiconductor surface chemistry for the formation of inorganic/organic interfaces with atomic precision. The setup used was designed for scientific investigations; without the analysis steps between each cycle, with a higher concentration of the reactants, etc., a faster reaction and much higher throughput is possible. As a major advantage, the cyclooctyne concept for the first layer is flexible with respect to the reactions applied for the attachment of further layers. This has been demonstrated for enol ether/tetrazine coupling; further reaction schemes such as azomethine ylide cycloadditions^75^ can be also envisioned. For stability of the layers under ambient conditions and/or further suppression of concomitant adsorption of oxygen on the surface, passivation of the unreacted dangling bonds remaining after the adsorption of the first layer would be necessary; strategies using smaller “inhibitor” molecules such as acetylene are under investigation.

Potential further steps in the development of this approach and future applications are shown in Figure 14. They might include cross-linking of the grown molecular chains (Figure 14(a)), e.g., using photochemistry as an additional parameter to achieve selectivity;^76^ the attachment of functional groups for sensing applications in a well-defined distance from the surface (Figure 14(b)), e.g., a pyrene as chromophore in the fourth layer such as **33** (Figure 14(f)); the well-controlled layered arrangement of molecules with given physical or chemical properties (Figure 14(c)); area-selective growth of molecular structures on surfaces,^12^ including, e.g., hydrogen desorption lithography^77,78^ (Figure 14(d)); or the application of covalent LBL growth on laterally structured surfaces, e.g., realized by means of on-surface chemistry (Figure 14(e)).

**Figure 14:**
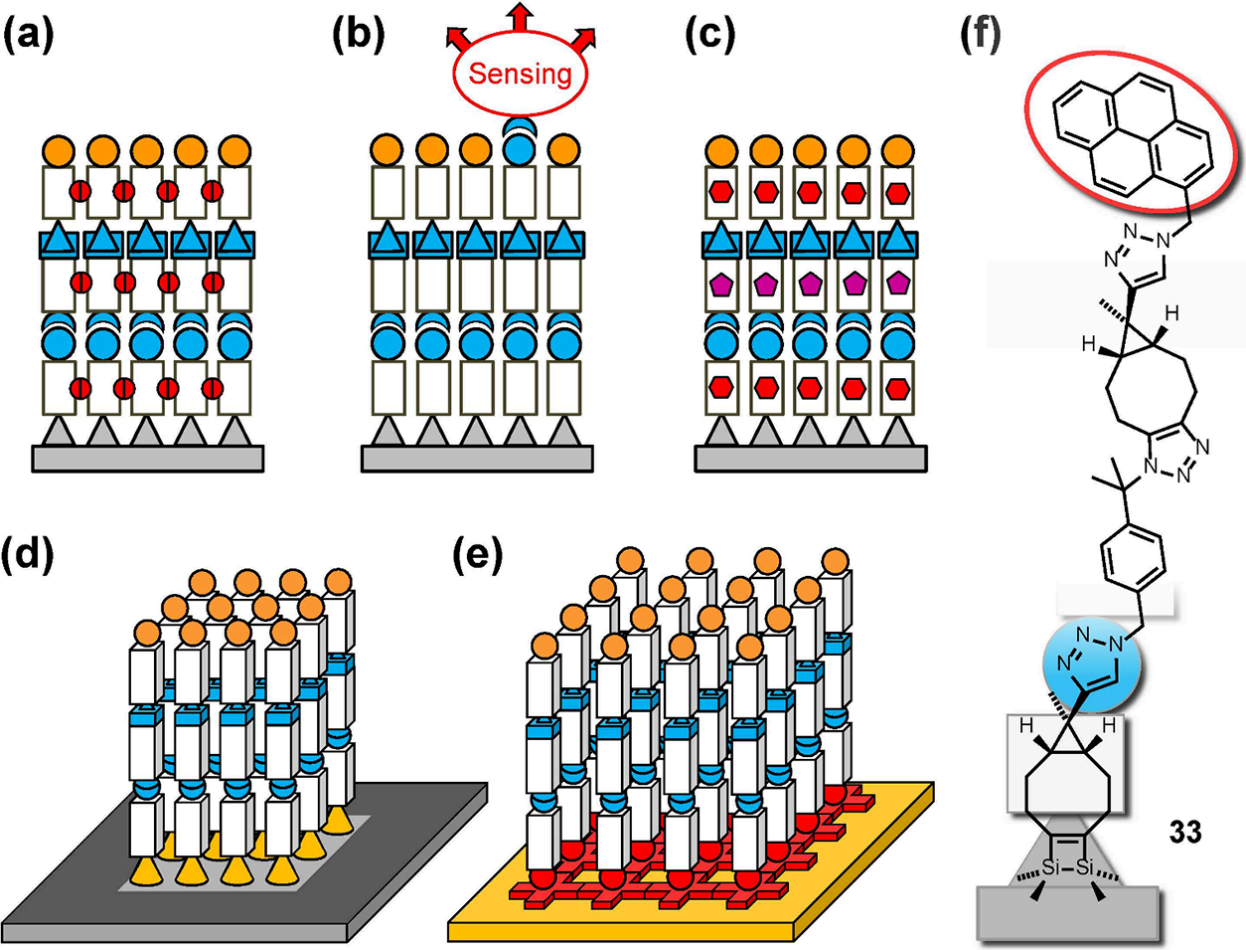
Potential developments in cyclooctyne-based covalent LBL growth.
